# Morphological and phylogenetic analyses revealed *Fissuracium ellipsoideum* gen. et sp. nov. (Amylocorticiaceae, Amylocorticiales) from southwest China

**DOI:** 10.3897/mycokeys.133.195561

**Published:** 2026-06-05

**Authors:** Changlin Zhao, Zirui Gu, Xiumei Xu, Xiangfu Liu

**Affiliations:** 1 Key Laboratory of Forest Resources Conservation and Utilization in the Southwest Mountains of China Ministry of Education, Modern Industry School of Edible-fungi, Southwest Forestry University, Kunming 650224, China Department Microbial Drugs (MWIS), Helmholtz-Centre for Infection Research Braunschweig Germany; 2 Department Microbial Drugs (MWIS), Helmholtz-Centre for Infection Research, 38124 Braunschweig, Germany College of Forestry, Southwest Forestry University Kunming China; 3 College of Forestry, Southwest Forestry University, Kunming 650224, China Modern Industry School of Edible-fungi, Southwest Forestry University Kunming China

**Keywords:** Fungal classification, molecular systematics, new taxa, wood-inhabiting fungi

## Abstract

Wood-inhabiting fungi play a fundamental role in ecosystem processes, particularly in wood degradation and the recycling of organic matter. In the present study, a new wood-inhabiting fungal genus *Fissuracium*, with its type species, *F. ellipsoideum* sp. nov., found in southwest China, is proposed based on a combination of morphological features and molecular evidence. The genus *Fissuracium* is characterized by the resupinate basidiomata with a cracking hymenophore, a monomitic hyphal system with clamped generative hyphae, the presence of cystidioles, and ellipsoid, thick-walled basidiospores. Sequences of the internal transcribed spacers (ITS), nuclear large subunit ribosomal RNA (nLSU), RNA polymerase second largest subunit (*rpb2*) and translation elongation factor 1-α (*tef1-α*) of the nuclear ribosomal DNA (rDNA) markers of the studied samples were generated. Phylogenetic analyses were performed using Maximum Likelihood, Maximum Parsimony, and Bayesian Inference. Multi-locus phylogenetic analyses of ITS+nLSU+*rpb2*+*tef1-α* showed that *Fissuracium* forms a monophyletic clade within the family Amylocorticiaceae, which was clustered into the order Amylocorticiales. The phylogenetic analyses indicated that the new genus *Fissuracium* formed a single lineage. A full description, illustrations, and phylogenetic analyses results of the new taxa are provided.

## Introduction

Wood-inhabiting fungi have significant industrial, edible, medicinal, nutritional, and other economic value (Wu et al. 2019; Ghobad-Nejhad et al. 2024; Hibbett et al. 2025; Ling et al. 2025). These fungi colonize diverse woody substrates, including living trees, standing deadwood, fallen logs, coarse branches, and stumps (Floudas and Hibbett 2015; Miettinen et al. 2016; Zhao et al. 2025). Wood-inhabiting fungi enzymatically degrade lignin, cellulose, and hemicelluloses through ligninolytic and cellulolytic enzymes, underscoring their critical role in organic matter recycling and nutrient cycling (Floudas and Hibbett 2015; Miettinen et al. 2016; Justo et al. 2017; James et al. 2020; Dai et al. 2021; Wu et al. 2022a; Wang et al. 2025).

Amylocorticiales comprises wood-inhabiting fungi, a small order comprising one family, 15 genera and 70 species (Larsson 2007; Binder et al. 2010; Dai 2011; He et al. 2024; Liu et al. 2026; Yuan et al. 2026). Morphologically, it is characterized by the resupinate corticioid basidiomata with smooth, merulioid, or poroid hymenophores, a monomitic hyphal system with loosely interwoven nodose-septate generative hyphae, the absence of cystidia, and smooth basidiospores with an amyloid reaction in Melzer’s reagent (Matheny et al. 2006; Bernicchia and Gorjón 2010; Binder et al. 2010). Amylocorticiales contains mostly resupinate forms with effused, effused-reflexed to almost pileate, or rarely multitiered pileate-stipitate basidiomata; the hymenial configuration varies from smooth to merulioid, irpicoid or poroid (Binder et al. 2010). Amyloid spore walls are not unique to Amylocorticiales and are widespread in both Russulales and Agaricales (Matheny et al. 2006; Bernicchia and Gorjón 2010; Chen et al. 2015; Zhou et al. 2025). Species in Amylocorticiales live saprotrophically on decaying wood or as plant parasites, in which they are normally associated with brown rot or, more rarely, with white rot (Binder et al. 2010).

The evolutionary relationship between Atheliales and Amylocorticiales within Agaricomycetidae, which is dominated by corticioid species, remains unclear (Matheny et al. 2006; He et al. 2024). Based on phylogenomic studies (Nagy et al. 2016), Atheliales is closely related to Amylocorticiales, but large-scale multigene phylogenies inferred from nuclear ribosomal SSU and LSU, 5.8S, *rpb1*, *rpb2*, and *tef1* showed that Amylocorticiales was most closely related to Agaricales, while Atheliales was closely related to Lepidostromatales, for which no genomes are currently available (Sulistyo et al. 2021). However, the order is well defined by gene and genomic analyses, although more genomes should be included to define the relationships of the different groups within Amylocorticiales. The phylogenetic study revealed relationships among the different groups of Agaricomycetidae, mainly Agaricales and Boletales, with the corticioid lineages containing predominantly resupinate forms (Binder et al. 2010). Several studies previously suggested that the Amylocorticiales should be considered within Agaricomycetidae, but their precise placement has not been clearly resolved (Binder et al. 2005; Hibbett and Binder 2002; Larsson et al. 2004; Larsson 2007). The analyses with nuc-lsu rRNA have placed the Amylocorticiales as the sister group of Agaricales or as the sister group of a clade containing Agaricales, Boletales and Atheliales (Binder et al. 2005; Hibbett and Binder 2002; Larsson et al. 2004; Larsson 2007). Analyses of Agaricales with nuc-lsu, nucssu and 5.8S rRNA genes, RNA polymerase II (rpb1, rpb2), suggested that the Amylocorticiales were the sister group of Agaricales, along with a clade containing certain clavarioid and pileate-stipitate agaricoid forms (Matheny et al. 2006).

The family Amylocorticiaceae Julich, belonging to the order Amylocorticiales (Basidiomycota), was typified by *Amylocorticium* Pouzar (Jülich 1982). Phylogenetically, only a few studies have been conducted, and the generic position of some lineages has not been resolved since the foundation of Amylocorticiaceae. Fifteen genera are recognized in the Amylocorticiaceae as *Agroathelia* Redhead & Mullineux, *Amyloathelia* Hjortstam & Ryvarden, *Amyloceraceomyces* S.H. He, *Amylocorticiellum* Spirin & Zmitr., *Amylocorticium* Pouzar, *Amylophanerochaete* Yu Q. Liu & S.H. He, *Amyloxenasma* (Oberw.) Hjortstam & Ryvarden, *Anomoloma* Niemelä & K.H. Larss., *Anomoporia* Pouzar, *Ceraceomyces* Jülich, *Plicatura* Peck, *Plicaturopsis* D.A. Reid, *Podoserpula* D.A. Reid, *Pseudoathelia* Yu Q. Liu & S.H. He and *Serpulomyces* Spirin & Zmitr. (Binder et al. 2010; Liu et al. 2026).

During investigations on wood-inhabiting fungi in the Yunnan-Guizhou Plateau, China many corticioid specimens were collected. To clarify the placement and relationships of these specimens, molecular phylogenetic and taxonomic studies were carried out on the order Amylocorticiales using combined ITS+nLSU+*rpb2*+*tef1-α* data analyses. Accordingly, *Fissuracium* gen. nov. and its type species *F. ellipsoideum* sp. nov. are formally described here, supported by morphological illustrations and multi-locus phylogeny.

## Materials and methods

### Sample collection and herbarium specimen preparation

The basidiomata were collected from fallen angiosperm branches in Yunnan Province, Southwest China. The samples were photographed *in situ*, and fresh macroscopic details and other important information were documented (Dong et al. 2024; Rathnayaka et al. 2025). Photographs were recorded by a Xiaomi 14 Ultra camera. Macroscopic observations were noted. Collected basidiomata were dried on an electric food dryer at 40 °C. Dried specimens were sealed in envelopes and zip-lock plastic bags and labeled with voucher numbers (Dong et al. 2025). The voucher specimens were deposited in the herbarium of the Southwest Forestry University (SWFC), Kunming, Yunnan Province, China.

### Morphological study

The macro-morphological descriptions were based on field notes and photos taken in the field and in the lab. The color terminology follows Petersen (1996) and was confirmed in general terms according to the CMYK color code (Deep White Printing Team 2022). The micro-morphological data were obtained from dried specimens observed under a light microscope at 10 × 100 magnification (Dong et al. 2024). Sections were mounted in 5% KOH and 2% Phloxine B (C_20_H_2_Br_4_C_l4_Na_2_O_5_) for microscopic observation. Cotton Blue and Melzer’s reagent were also used to examine micromorphological structures. Congo red was used as a stain when necessary (Horak 2005). To show the variation in spore sizes, 5% of measurements were excluded from each end of the range and shown in parentheses. At least 30 basidiospores from each specimen were measured. The following abbreviations are used: KOH = 5% potassium hydroxide water solution, CB– = acyanophilous, CB+ = cyanophilous, IKI– = both inamyloid and indextrinoid, L = mean spore length (arithmetic average for all spores), W = mean spore width (arithmetic average for all spores), Q = variation in the L/W ratios between the specimens studied, Qm = mean Q value ± standard deviation, and n = a/b (number of spores (a) measured from given number (b) of specimens).

### DNA extraction, PCR amplification, sequencing and phylogenetic analyses

The CTAB rapid plant genome extraction kit-DN14 (Aidlab Biotechnologies Co., Ltd, Beijing, China) was used to obtain genomic DNA from the dried fungal specimens according to the manufacturer’s instructions (Dong et al. 2024; Yang et al. 2025). The extracted DNA was maintained at –20 °C for long-term storage. Four molecular markers were investigated, i.e., the internal transcribed spacer (ITS), the nuclear large subunit ribosomal RNA (nLSU), the RNA polymerase II subunit 2 (*rpb*2) gene, and the translation elongation factor 1-α (*tef*1-α) gene. The primers and conditions are shown in Table 1. The PCR products were purified and sequenced at Kunming Tsingke Biological Technology Limited Company (Yunnan Province, China). All newly generated sequences were deposited in NCBI GenBank (https://www.ncbi.nlm.nih.gov/genbank/) (Table 2).

**Table 1.** Loci, primers, PCR amplification procedures, and references used in this study.

Locus name	Abbreviation	Name	Direction	Sequence (5'-3')	PCR amplification procedures	References
Internal transcribed spacer region of the rDNA	ITS	ITS5	Forward	GGAAGTAAAAGTCGTAACAAGG	94 °C 2 min; 35 cycles of 94 °C 60 s, 55 °C 60 s, 72 °C 2 min; 72 °C 10 min.	White et al. (1990)
		ITS4	Reverse	TCCTCCGCTTATTGATATGC		
Nuclear large subunit ribosomal	nLSU	LR0R	Forward	ACCCGCTGAACTTAAGC	94 °C 2 min; 35 cycles of 94 °C 30 s, 48 °C 1 min, 72 °C 1.5 min; 72 °C 10 min.	Vilgalys and Hester (1990)
		LR7	Reverse	TACTACCACCAAGATCT		
RNA polymerase second largest subunit	*rpb2*	RPB2-6F	Forward	TGGGGYATGGTNTGYCCYGC	94 °C 2 min; 9 cycles of 94 °C 45 s, 60 °C 45 s, 72 °C 1.5 min; 36 cycles of 94 °C 45 s, 53 °C 1 min, 72 °C 1.5 min; 72 °C 10 min.	Liu et al. (1999)
		RPB2-7cR	Reverse	CCCATRGCTTGYTTRCCCAT		
Translation elongation factor 1-α	*tef1-*α	EF1-983 F	Forward	GCYCCYGGHCAYCGTGAYTTYAT	94 °C 1 min; 35 cycles of 94 °C 30 s, 59 °C 1 min, 72 °C 1.5 min; 72 °C 10 min.	Rehner and Buckley (2005)
		EF1-2218R	Reverse	ATGACACCRACRGCRACRGTYTG		

**Table 2.** A list of species, specimens and GenBank accession numbers of sequences used in this study. [New species is shown in bold; * refers to the type species, ^T^ refers to the type specimen and — refers to the missing data.]

**Species nam**e	**Specimen No**.	**GenBank accession No**.				**Country**	**References**
		**ITS**	**nLUS**	***rpb2***	***tef1-α***		
Agroathelia rolfsii*	AFTOL-ID 664	—	AY635773	—	—	USA	—
Agroathelia rolfsii*	ATCC 201126	AF499018	AF499019	—	—	Argentina	—
*Amphinema byssoides*	EL 11/98	AY463375	AY586626	—	—	Estonia	Larsson et al. (2004)
*Amphinema byssoides*	M. Ryberg	GQ162810	GQ162810	—	—	Sweden	Kotiranta et al. (2011)
*Amyloathelia crassiuscula*	GB/K 169-796	DQ144610	—	—	—	Sweden	Yuan et al. (2020)
Amyloceraceomyces angustisporus*	He 2819	MK520871	—	—	—	China	Yuan et al. (2020)
Amyloceraceomyces angustisporus*	He 2824	MK520872	MK491337	—	—	China	Yuan et al. (2020)
Amyloceraceomyces angustisporus*	He 2844 ^T^	MK520873	MK491338	—	—	China	Bernicchia and Gorjón (2010)
Amylocorticiellum subillaqueatum*	KHL 8493	AY463431	AY586679	—	—	Sweden	Larsson et al. (2004)
*Amylocorticium cebennense*	CFMR:HHB-2808	GU187505	GU187561	GU187770	GU187675	USA	Binder et al. (2010)
*Amylocorticium cebennense*	He 3074	PV185711	—	—	—	China	Liu et al. (2026)
*Amylocorticium ellipsosporum*	He 4457 ^T^	MK520876	MK491341	—	—	China	Yuan et al. (2020)
*Amylocorticium subincarnatum*	AS 95	AY463377	AY586628	—	—	Sweden	Larsson et al. (2004)
Amylocorticium subsulphureum*	CFMR:HHB-13817	GU187506	GU187562	GU187773	GU187680	USA	Binder et al. (2010)
Amylophanerochaete hainanense*	He 7950 ^T^	PV185713	PV211328	—	—	China	Liu et al. (2026)
Amyloxenasma allantosporum*	SREF 166	MN660447	—	—	—	Poland	Maunoury et al. (2020)
Amyloxenasma allantosporum*	SREF 408	MN660833	—	—	—	Poland	Maunoury et al. (2020)
Amyloxenasma allantosporum*	KHL s.n.	GU187498	GU187666	—	—	Sweden	Yuan et al. (2020)
Anomoloma albolutescens*	LYBR 5671	ON053465	ON038410	—	—	China	Yuan et al. (2020)
Anomoloma albolutescens*	CFMR:L-6088	GU187507	GU187563	GU187768	—	USA	Binder et al. (2010)
*Anomoloma luteoalbum*	Cui 2687 ^T^	KT954961	KT954975	—	—	China	Song et al. (2016)
*Anomoloma luteoalbum*	Cui 8686	KT954962	KT954976	—	—	China	Song et al. (2016)
*Anomoloma myceliosum*	CFMR:MJL-4413	GU187500	GU187559	GU187766	—	Canada	Binder et al. (2010)
*Anomoloma myceliosum*	JV0509/117	OM914151	—	—	—	China	Zhou et al. (2022)
*Anomoloma rhizosum*	Cui 9717	KT954958	KT954972	—	—	China	Song et al. (2016)
*Anomoloma rhizosum*	Cui 10589	KT954959	KT954973	—	—	China	Song et al. (2016)
Anomoporia bombycina*	CFMR:L-6240	GU187508	GU187564	GU187765	—	USA	Binder et al. (2010)
*Anomoporia kamtschatica*	KHL 11072	—	AY586630	—	—	Sweden	Larsson et al. (2004)
*Anomoporia kamtschatica*	GB/M EdmanK426	—	DQ144615	—	—	Sweden	—
*Anomoporia vesiculosa*	Cui 9523	KT954950	—	—	—	China	Song et al. (2016)
*Anomoporia vesiculosa*	Dai 22795	ON413718	ON413720	—	—	China	Yuan et al. (2020)
*Anthracophyllum archeri*	AFTOL 973	DQ404387	AY745709	DQ385877	DQ028586	USA	Matheny et al. (2006)
*Aphanobasidium pseudotsugae*	HHB 822	GU187509	GU187567	GU187781	GU187695	USA	Binder et al. (2010)
*Athelia arachnoidea*	CBS 418.72	GU187504	GU187557	GU187769	GU187672	Netherlands	Binder et al. (2010)
*Athelia arachnoidea*	GB 0087426	LR694192	LR694169	LR694267	LR694213	Sweden	Sulistyo et al. (2021)
*Auricularia polytricha*	TUFC 12920	AB871752	AB871733	—	—	Japan	Sotome et al. 2014
*Auricularia tibetica*	Dai 13336	MZ618943	MZ669915	—	—	China	Miettinen et al. (2012)
Boletopsis leucomelaena*	AFTOL 1527	DQ484064	DQ154112	GU187820	GU187763	USA	Binder et al. (2010)
Bondarzewia montana*	AFTOL 452	DQ200923	DQ234539	AY218474	DQ059044	Canada	Matheny et al. (2007)
*Byssocorticium atrovirens*	BS 1710033	LR694198	LR694175	LR694271	LR694214	Sweden	Sulistyo et al. (2021)
*Byssocorticium atrovirens*	GB 0078129	LR694199	LR694176	LR694272	LR694215	Sweden	Sulistyo et al. (2021)
Byssoporia terrestris*	Hjm 18172	EU118608	EU118608	—	—	Sweden	Larsson (2007)
Byssoporia terrestris*	GB 0058650	—	LR694178	—	—	Sweden	Sulistyo et al. (2021)
*Calocera cornea*	AFTOL 438	AY789083	AY701526	AY536286	AY881019	USA	Hanif and Khalid (2015)
*Ceraceomyces atlanticus*	URM 85888 ^T^	KX685875	KX685874	—	—	Brazil	Chikowski et al. (2016)
Ceraceomyces tessulatus*	He 3008	PV185714	PV211329	—	—	China	Liu et al. (2026)
Ceraceomyces tessulatus*	KHL 16429	KU518951	KU518951	—	—	Norway	Chikowski et al. (2016)
Chondrostereum purpureum*	AFTOL 441	DQ200929	AF518607	AY218477	DQ457632	USA	Matheny et al. (2006)
*Coltricia perennis*	AFTOL 447	DQ234559	AF287854	AY218526	AY885147	USA	Wang et al. (2004)
*Coniophora arida*	FP 104367	GU187510	GU187573	GU187775	GU187684	USA	Binder et al. (2010)
*Coniophora marmorata*	MUCL 31667	GU187515	GU187571	GU187780	GU187688	Belgium	Binder et al. (2010)
Digitatispora marina*	3027C	KM272371	KM272362	—	—	Norway	Rämä et al. (2014)
Echinodontium tinctorium*	AFTOL 455	AY854088	AF393056	AY218482	AY885157	USA	Sulistyo et al. (2021)
Fissuracium ellipsoideum*	**CLZhao 34498**	**PX965999**	**PX966001**	**PZ284856**	**PZ284858**	**China**	**Present Study**
***Fissuracium ellipsoideum*** *	**CLZhao 34584 ^T^**	**PX966000**	**PX966002**	**PZ284857**	**PZ284859**	**China**	**Present Study**
*Fomitiporia gabonensis*	MUCL 47576	GU461971	GU461990	JQ087972	GU461923	Gabon	Amalfi et al. (2012)
*Fomitiporia mediterranea*	AFTOL 488	AY854080	AY684157	AY803748	AY885149	USA	Sulistyo et al. (2021)
*Fomitiporia sonorae*	MUCL 47689	JQ087893	JQ087920	JQ088006	JQ087947	USA	Amalfi et al. (2012)
Fomitopsis pinicola*	AFTOL 770	AY854083	AY684164	AY786056	AY885152	USA	Sulistyo et al. (2021)
*Ganoderma tsugae*	AFTOL 771	DQ206985	AY684163	DQ408116	DQ059048	USA	Matheny et al. (2007)
*Gautieria otthii*	AFTOL 466	AF377072	AF393058	AY218486	AY883434	USA	Wang et al. (2004)
*Gloeophyllum striatum*	ARIZ AN027866	HM536092	HM536063	HM640259	HM536111	USA	Garcia-Sandoval et al. (2011)
*Gloeophyllum trabeum*	1320	HM536094	HM536067	HM536112	HM536113	USA	Garcia-Sandoval et al. (2011)
*Gomphidius roseus*	AFTOL 1780	DQ534570	DQ534669	GU187818	GU187702	Germany	Binder et al. (2010)
Grifola frondosa*	AFTOL 701	AY854084	AY629318	AY786057	AY885153	USA	Sulistyo et al. (2021)
*Gymnopilus picreus*	ZRL 2015011	LT716066	KY418882	KY419027	KY419077	China	Zhao et al. (2017)
*Gyrodontium sacchari*	MUCL 40589	GU187522	GU187579	GU187764	GU187703	French Guiana	Binder et al. (2010)
Heliocybe sulcata*	IBUG 9930	HM536095	HM536069	HM536114	HM536115	Mexico	Garcia-Sandoval et al. (2011)
*Henningsomyces candidus*	AFTOL 468	AY571043	AF287864	AY218513	AY883424	Canada	Hibbett et al. (2000)
Heterobasidion annosum*	AFTOL 470	DQ206988	AF287866	AH013701	DQ028583	USA	Matheny et al. (2007)
*Hydnellum geogenium*	AFTOL 680	DQ218304	AY631900	DQ408133	DQ059053	USA	Matheny et al. (2007)
Hydnomerulius pinastri*	MD 312	GU187523	GU187580	GU187787	GU187708	USA	Binder et al. (2010)
*Hydnum albomagnum*	AFTOL 471	DQ218305	AY700199	DQ234553	DQ234568	USA	Matheny et al. (2007)
Jaapia argillacea*	CBS 252.74	GU187524	GU187581	GU187788	GU187711	Netherlands	Binder et al. (2010)
Jaapia argillacea*	CBS 252.74	NR119766	NG042523	—	—	USA	Binder et al. (2010)
*Jaapia ochroleuca*	KHL 8433	EU118637	EU118637	—	—	Sweden	Larsson (2007)
*Lactarius deceptivus*	AFTOL 682	AY854089	AY631899	AY803749	AY885158	USA	Sulistyo et al. (2021)
*Lactarius lignyotus*	AFTOL 681	DQ221107	AY631898	DQ408128	DQ435787	USA	Matheny et al. (2007)
*Lepidostroma vilgalysii*	RV MX16	JN698907	JN698908	—	—	Mexico	Hodkinson et al. (2012)
*Lepiota cristata*	ZRL 20151133	LT716026	KY418841	KY418992	KY419048	China	Zhao et al. (2017)
Lepista irina	AFTOL 815	DQ221109	DQ234538	DQ385885	DQ028591	USA	Matheny et al. (2007)
Mythicomyces corneipes*	AFTOL 972	DQ404393	AY745707	DQ408110	DQ029197	Germany	Tang et al. (2024)
*Neolentinus adhaerens*	DAOM 214911	HM536096	HM536071	HM536116	HM536117	USA	Garcia-Sandoval et al. (2011)
*Oligoporus rennyi*	KEW 57	AY218416	AF287876	AY218499	—	USA	Wang et al. (2004)
*Penttilamyces lichenicola*	DAOM 194172	GU187531	GU187583	GU187789	GU187715	Canada	Binder et al. (2010)
*Penttilamyces olivascens*	HHB 11134	GU187532	GU187587	GU187790	GU187717	USA	Binder et al. (2010)
Phallus hadriani	AFTOL 683	DQ404385	AY885165	DQ408114	DQ435792	USA	Sulistyo et al. (2021)
Phlebia radiata*	AFTOL 484	AY854087	AF287885	AY218502	AY885156	USA	Hibbett et al. (2000)
*Piloderma byssinum*	GB 0121002	LR694206	LR694184	LR694279	—	Sweden	Sulistyo et al. (2021)
*Piloderma byssinum*	KHL 8456	AY463453	AY586699	—	—	Sweden	Larsson et al. (2004)
*Plicatura nivea*	CBS 482.72	MH860536	MH872242	—	—	Canada	Vu et al. (2019)
*Plicatura pendula*	BPalla 19111526	PQ722793	PQ721674	PQ678964	—	Hungary	Palla et al. (2024)
*Plicatura pendula*	GB/B.Norden	DQ144619	DQ144619	—	—	Sweden	Chikowski et al. (2016)
Plicaturopsis crispa*	CFMR:DLL2011-011	KJ140537	—	—	—	USA	Brazee et al. (2014)
Plicaturopsis crispa*	HMAS 293434	OR237017	—	—	—	China	—
Plicaturopsis crispa*	LWZ (2020)1017-11	ON897938	ON885398	—	—	China	Yuan et al. (2020)
Plicaturopsis crispa*	MR 00464	LR694209	LR694187	LR694281	LR694225	Sweden	Sulistyo et al. (2021)
*Podoserpula ailaoshanensis*	Liu 170	KU324485	KU324488	—	—	China	Zhou et al. (2016)
*Podoserpula ailaoshanensis*	ZJL 2015015 ^T^	KU324484	KU324487	—	—	China	Zhou et al. (2016)
Podoserpula pusio*	H. Lepp 329 ACT	GU187555	—	—	—	USA	Binder et al. (2010)
Podoserpula pusio*	PDD 81253	MN970537	—	—	—	Chile	Garnica et al. (2021)
Podoserpula pusio*	AFTOL 1522	DQ494688	DQ470821	—	—	Australia	Yuan et al. (2020)
*Pseudoathelia linzhiense*	CLZhao 31183 ^T^	NR198738	PP862918	—	—	China	Zhou et al. (2024)
*Pseudoathelia linzhiense*	CLZhao 31174	PP399152	PP862922	—	—	China	Zhou et al. (2024)
*Pseudoathelia septentrionalis*	UC 2023047	KP814348	—	—	—	USA	Rosenthal et al. (2017)
Pseudomerulius aureus*	FP 103859	GU187534	GU187590	GU187799	GU187731	USA	Binder et al. (2010)
*Pseudomerulius curtisii*	REH 8912	GU187533	GU187589	GU187796	GU187725	Australia	Binder et al. (2010)
*Punctularia strigosozonata*	AFTOL 1248	DQ398958	AF518642	DQ381843	DQ408147	USA	Hibbett and Binder (2002)
Resinicium bicolor*	AFTOL 810	DQ218310	AF393061	DQ457635	DQ061277	USA	Matheny et al. (2007)
Rickenella fibula*	AFTOL 486	DQ241782	AY700195	DQ408115	DQ435794	USA	Gérald et al. (2017)
*Sebacina schweinitzii*	AFTOL 699	DQ411526	AY745701	DQ408132	DQ029196	USA	Sulistyo et al. (2021)
*Serendipita indica*	AFTOL 612	DQ411527	AY293202	DQ408131	AJ249911	USA	Binder et al. (2005)
Serpulomyces borealis*	CFMR: L-8014	GU187512	GU187570	—	—	USA	Binder et al. (2010)
*Serpulomyces rhizomorphus*	CLZhao 31188 ^T^	NR198737	NG244072	—	—	China	Zhou et al. (2024)
*Serpulomyces rhizomorphus*	CLZhao 31197	PP399150	PP862916	—	—	China	Zhou et al. (2024)
*Serpulomyces yunnanensis*	CLZhao 18992	OQ132519	OQ147003	—	—	China	Yuan et al. (2023)
Sparassis crispa*	AFTOL 703	DQ250597	AY629321	DQ408122	DQ056289	China	Matheny et al. (2007)
*Steccherinum tenue*	KHL 12316	JN710598	JN710598	JN710739	JN710733	USA	Miettinen et al. (2012)
*Stereopsis globosa*	KHL 12592	KC203495	KC203495	KC203501	KC203515	Costa Rica	Sjökvist et al. (2014)
Stereopsis radicans*	LR 45395	KC203496	KC203496	KC203502	KC203516	Belize	Sjökvist et al. (2014)
Sulzbacheromyces caatingae*	Sulzbacher 1479	KC170320	KC170318	—	—	Brazil	Sulzbacher et al. (2012)
*Trametes versicolor*	ZRL 20151477	LT716079	KY418903	KY419041	KY419091	China	Zhao et al. (2017)
*Trechispora farinacea*	KHL 8451	AF347082	AF347082	—	—	Sweden	Larsson et al. (2004)
*Trechispora hymenocystis*	KHL 8795	AF347090	AF347090	—	—	Sweden	Larsson et al. (2004)
Vuilleminia comedens*	AFTOL 1247	DQ398959	AF518666	DQ381844	—	USA	Hibbett and Binder (2002)

**Figure 1.**
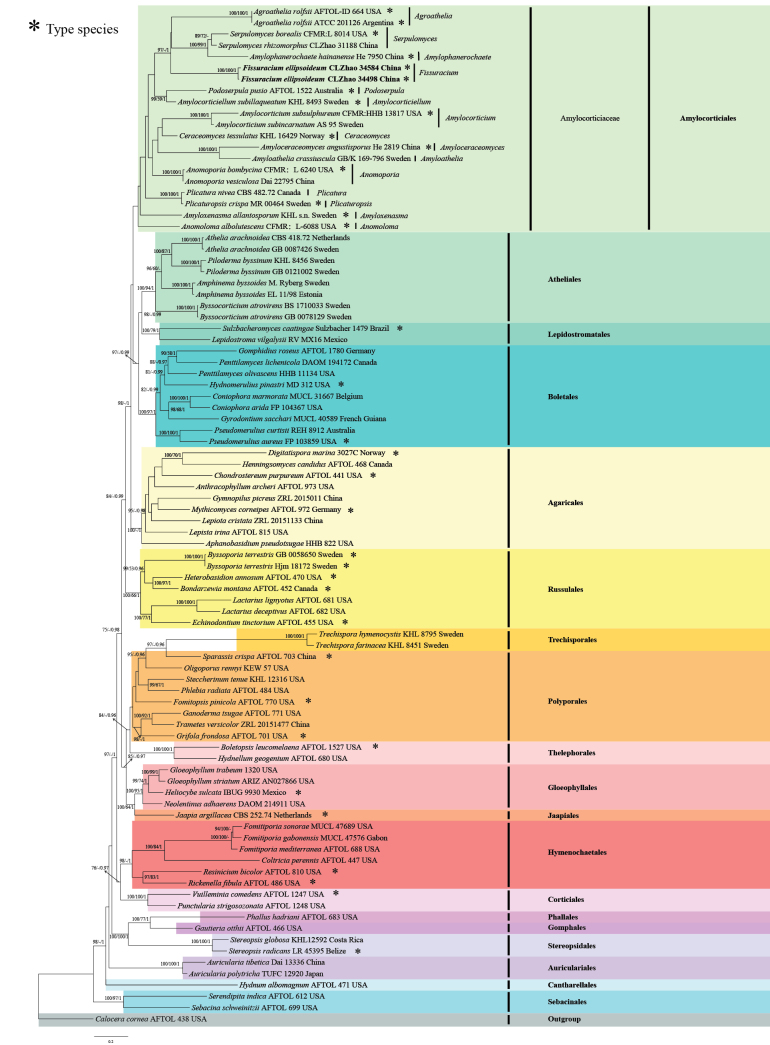
Maximum Likelihood analysis illustrating the phylogeny of *Fissuracium* and related genera in the order Amylocorticiales based on ITS+nLSU+*rpb2*+*tef1-α* sequences. Branches are labelled with maximum likelihood bootstrap value ≥ 70%, parsimony bootstrap value ≥ 50%, and Bayesian posterior probabilities ≥ 0.95. Colored bars represent different genera. * refers to type material (holotype).

**Figure 2.**
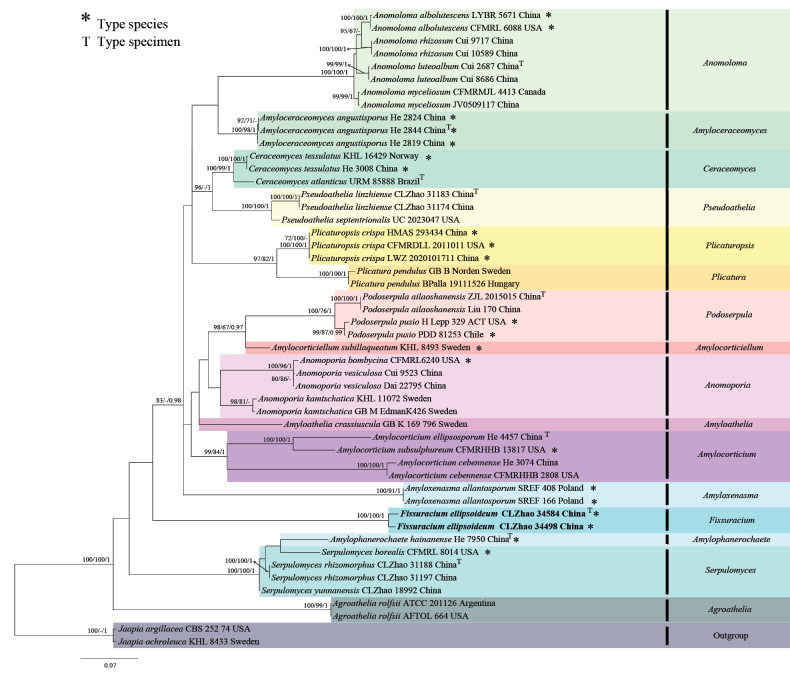
Maximum Likelihood analysis illustrating the phylogeny of *Fissuracium* and related genera in the family Amylocorticiaceae based on ITS+nLSU+*rpb2*+*tef1-α* sequences. Branches are labelled with maximum likelihood bootstrap value ≥ 70%, parsimony bootstrap value ≥ 50%, and Bayesian posterior probabilities ≥ 0.95. Colored bars represent different genera. New species are shown in bold, “T” refers to the generic species of genus, * refers to type material (holotype).

Sequences generated for this study were aligned, with additional sequences downloaded from GenBank. Sequences were aligned in MAFFT 7 (https://mafft.cbrc.jp/alignment/server/), adjusting the direction of nucleotide sequences according to the first sequence (accurate enough for most cases), and selecting the G-INS-i iterative refinement method (Katoh et al. 2019). The alignment was adjusted manually using AliView version 1.27 (Larsson 2014). The dataset was first aligned, and then ITS+nLSU+*rpb*2+*tef*1-α sequences were combined in Mesquite v. 3.81. The combined ITS+nLSU+*rpb2*+*tef1*-*α* dataset was used to infer phylogenies of the new genus and related species within Amylocorticiaceae. For the analysis, *Calocera cornea* (Batsch) Fr. was used as the outgroup for phylogenetic analysis (Fig. 1; Sulistyo et al. 2021). *Jaapia argillacea* Bres. and *Jaapia ochroleuca* (Bres.) Nannf. & J. Erikss. were used as outgroup for phylogenetic analysis of ITS+nLSU+*rpb2*+*tef1-α* phylogenetic tree (Fig. 2; Liu et al. 2026).

Maximum Parsimony (MP), Maximum Likelihood (ML), and Bayesian Inference (BI) analyses were applied these three methods to the combined datasets following the previous studies (Yang et al. 2025). Maximum Parsimony (MP) analysis was performed in PAUP* v. 4.0b10 (Swofford 2002). All characters were equally weighted, and gaps were treated as missing data. Trees were inferred using the heuristic search option with TBR branch swapping and 1,000 random sequence additions. Max trees were set to 5,000, branches of zero length were collapsed, and all parsimonious trees were saved. Clade robustness was assessed using bootstrap (BT) analysis with 1,000 replicates (Felsenstein 1985). Descriptive tree statistics, tree length (TL), consistency index (CI), retention index (RI), rescaled consistency index (RC), and homoplasy index (HI) were calculated for each maximum parsimonious tree generated. Maximum likelihood (ML) analysis was performed with RAxML-HPC BlackBox in CIPRES Science Gateway (https://www.phylo.org/portal2/login!input.action, Miller et al. 2012) using a GTRCAT model of evolution with 1,000 bootstrap replicates. ModelFinder v2.2.0 (Kalyaanamoorthy et al. 2017) was used to select the best-fit model using Bayesian Information Criterion (BIC). Bayesian Inference (BI) phylogenies were inferred with PhyloSuite v1.2.3 (Zhang et al. 2020; Xiang et al. 2023) and MrBayes v3.2.7a (Ronquist et al. 2012). Branches were considered significantly supported if they received a Maximum Likelihood bootstrap value (BS) of ≥ 70%, a Maximum Parsimony bootstrap value (BT) of ≥ 50%, or Bayesian Posterior Probabilities (BPP) of ≥ 0.95.

## Results

### Phylogenetic analyses

In the order Amylocorticiales analyses, the combined ITS+nLSU+*rpb2*+*tef1-α* dataset (Fig. 1) included sequences from 90 fungal specimens representing 83 taxa. The dataset had an aligned length of 6,250 characters, of which 2,994 characters are constant, 780 are variable and parsimony-uninformative, and 2,476 are parsimony-informative, in which the different partitions are as follows: ITS (2,654 bp), nLSU (1,876 bp), *rpb2* (912 bp), *tef1-α* (1,166 bp). Maximum parsimony analysis yielded 2 equally parsimonious trees (TL = 28,403, CI = 0.2243, HI = 0.7757, RI = 0.2796, and RC = 0.0627). The RAxML analysis of the combined dataset yielded a best-scoring tree (Fig. 1), with a final ML optimisation likelihood value of -99886.390. The alignment spanned 6,250 bp. The matrix had 3,653 distinct alignment patterns. Estimated base frequencies; A = 0.248, C = 0.231, G = 0.270, T = 0.251; substitution rates AC = 1.41721, AG = 4.04841, AT = 1.82375, CG = 1.00742, CT = 7.56987, GT = 1.00000. The best model of BI for the ITS+nLSU+*rpb2*+*tef1-α* dataset was GTR+F+I+G4. The average standard deviation of split frequencies in the Bayesian analyses reached 0.007931 (BI), and the effective sample size (ESS) for the Bayesian analysis across the two runs was double the average ESS (avg ESS) = 2,064.8. Branches that received bootstrap support for ML and BI ≥ 70% and 0.95, respectively, were considered significantly supported. The topology based on ITS+nLSU+*rpb2*+*tef1-α* sequences (Fig. 1) showed that the new taxa were clustered into the order Amylocorticiales.

In the family Amylocorticiaceae analyses, the ITS+nLSU+*rpb2*+*tef1-α* dataset (Fig. 2) included sequences from 50 fungal specimens representing 30 taxa. The dataset had an aligned length of 3,986 characters, of which 2,609 characters are constant, 431 are variable and parsimony-uninformative, and 946 are parsimony-informative, in which the different partitions are as follows: ITS (873 bp), nLSU (1,395 bp), *rpb2* (864 bp), *tef1-α* (848 bp). Maximum parsimony analysis yielded 150 equally parsimonious trees (TL = 3,637, CI = 0.5557, HI = 0.4443, RI = 0.7061, and RC = 0.3924). The RAxML analysis of the combined dataset yielded a best-scoring tree (Fig. 2), with a final ML optimisation likelihood value of -19513.515. The alignment contained 3,986 bp. The matrix had 1,295 distinct alignment patterns. Estimated base frequencies; A = 0.258, C = 0.211, G = 0.268, T = 0.263; substitution rates AC = 1.47354, AG = 3.66928, AT = 2.06321, CG = 0.63903, CT = 7.65684, GT = 1.00000. The best model of BI for the ITS+nLSU+*rpb2*+*tef1-α* dataset was GTR+F+I+G4. The average standard deviation of split frequencies in the Bayesian analyses was 0.007286 (BI), and the effective sample size (ESS) for the Bayesian analysis across the two runs was double the average ESS (avg ESS) = 1,800.4. Branches that received bootstrap support for ML and BI ≥ 70% and 0.95, respectively, were considered significantly supported. The topology based on the concatenated dataset (Fig. 2) showed that the new taxon was clustered within Amylocorticiaceae, forming a distinct lineage. Accordingly, we introduce *Fissuracium* gen. nov., with *F. ellipsoideum* sp. nov. designated as the type species.

The application of the PHI test to the ITS tree-locus sequences revealed no evidence of recombination among phylogenetically related species. No significant recombination events were observed among *Fissuracium ellipsoideum* and phylogenetically closely related species (Fig. 6). The test results for the ITS sequence dataset show that Φw = 0.90 (Φw > 0.05), indicating no recombination among the new species and related close species.

### Taxonomy

#### Fissuracium

Taxon classificationFungiAmylocorticialesAmylocorticiaceae

C.L. Zhao gen. nov.

BE6354B7-14D9-5465-8A5F-64FB2C30FFC1

863469

##### Chinese name.

硬撕裂革菌属 (ying si lie ge jun shu).

##### Etymology.

*Fissuracium* (Lat.): refers to the cracking basidiomata.

##### Description.

Basidiomata annual, resupinate, adnate, membranaceous, without odor or taste when fresh, becoming fragile upon drying. Hymenial surface smooth, cream when fresh, pale yellow when dry, obviously cracking. Hyphal system monomitic; generative hyphae with clamp connections, colorless, thin-walled. Basidia more or less pyriform, with four sterigmata and a basal clamp connection. Basidiospores ellipsoid, colorless, thick-walled, smooth, IKI–, slightly CB+. Causing a white rot.

##### Type species.

*Fissuracium ellipsoideum* C.L. Zhao.

##### Notes.

In our analyses, *Fissuracium* forms a distinct clade typified by *F. ellipsoideum*. The new genus was placed within Amylocorticiaceae (Amylocorticiales) and then grouped with *Amylophanerochaete* and *Serpulomyces* (Figs1 and 2). However, morphologically, *Amylophanerochaete* differs from *Fissuracium* by having the pellicular to membranaceous basidiomata with rhizomorphs and amyloid basidiospores (Liu et al. 2026). *Serpulomyces* differs from *Fissuracium* by having merulioid hymenophores and cylindrical to fusiform basidiospores (Zmitrovich and Spirin 2002).

#### Fissuracium ellipsoideum

Taxon classificationFungiAmylocorticialesAmylocorticiaceae

C.L. Zhao sp. nov.

914E32E8-E7A6-5A8F-9F52-D42EBCDDAD12

863470

Figs 3, 4, 5, 6

##### Chinese name.

椭圆孢硬撕裂革菌 (tuo yuan bao ying si lie ge jun).

##### Holotype.

China • Yunnan Province: Diqing, Weixi County, Weideng Town, Songpo Village, GPS coordinates: 27°5'N, 99°13'E, elevation: 1,600 m asl., on the fallen angiosperm branch, leg. C.L. Zhao, 13 October 2023, CLZhao 34584 (SWFC 00034584).

**Figure 3.**
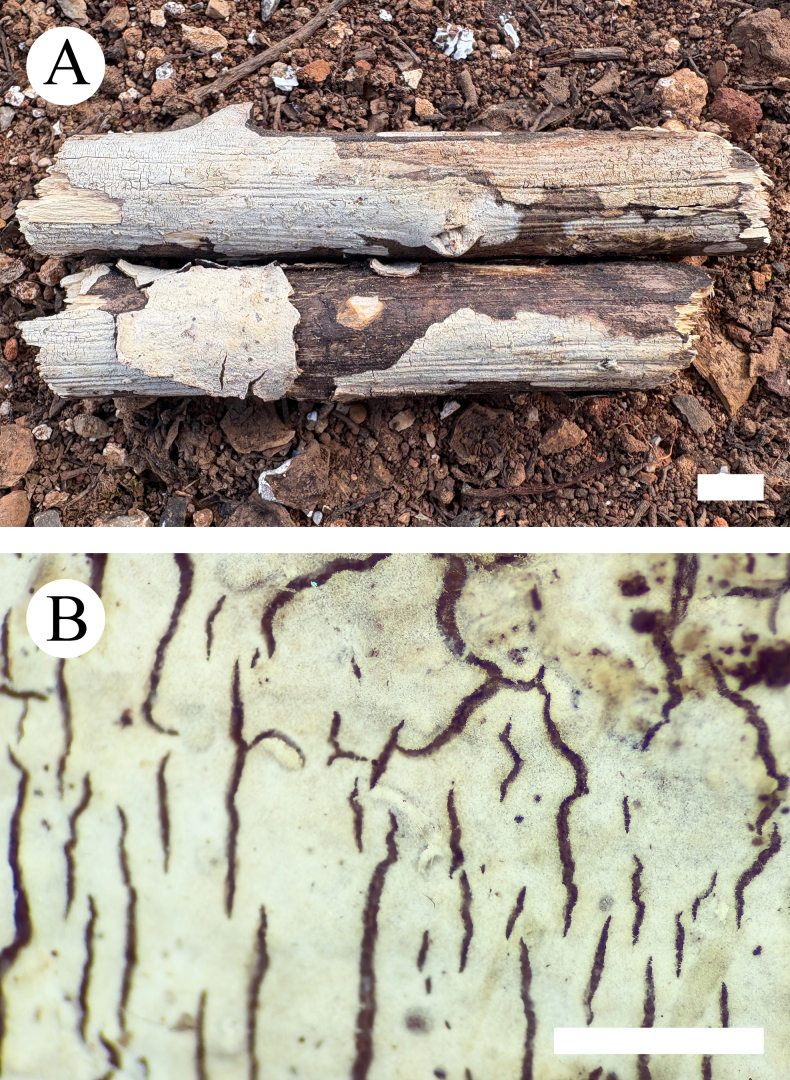
*Fissuracium ellipsoideum* (holotype, CLZhao 34584). **A**. Basidiomata on the substrate; **B**. Macroscopic characteristics of hymenophore. Scale bars: 1 cm (**A**); 1 mm (**B**).

**Figure 4.**
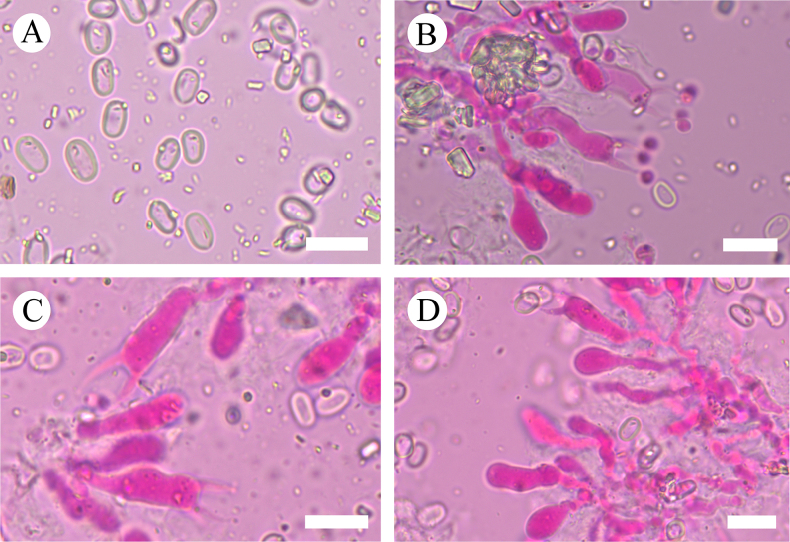
Sections of hymenium of *Fissuracium ellipsoideum* (holotype, CLZhao 34584). **A**. Basidiospores; **B, C**. Basidia and basidioles; **D**. Part of the generative hyphae. Scale bars: 10 μm (**A–D**); 10 × 100 Oil.

**Figure 5.**
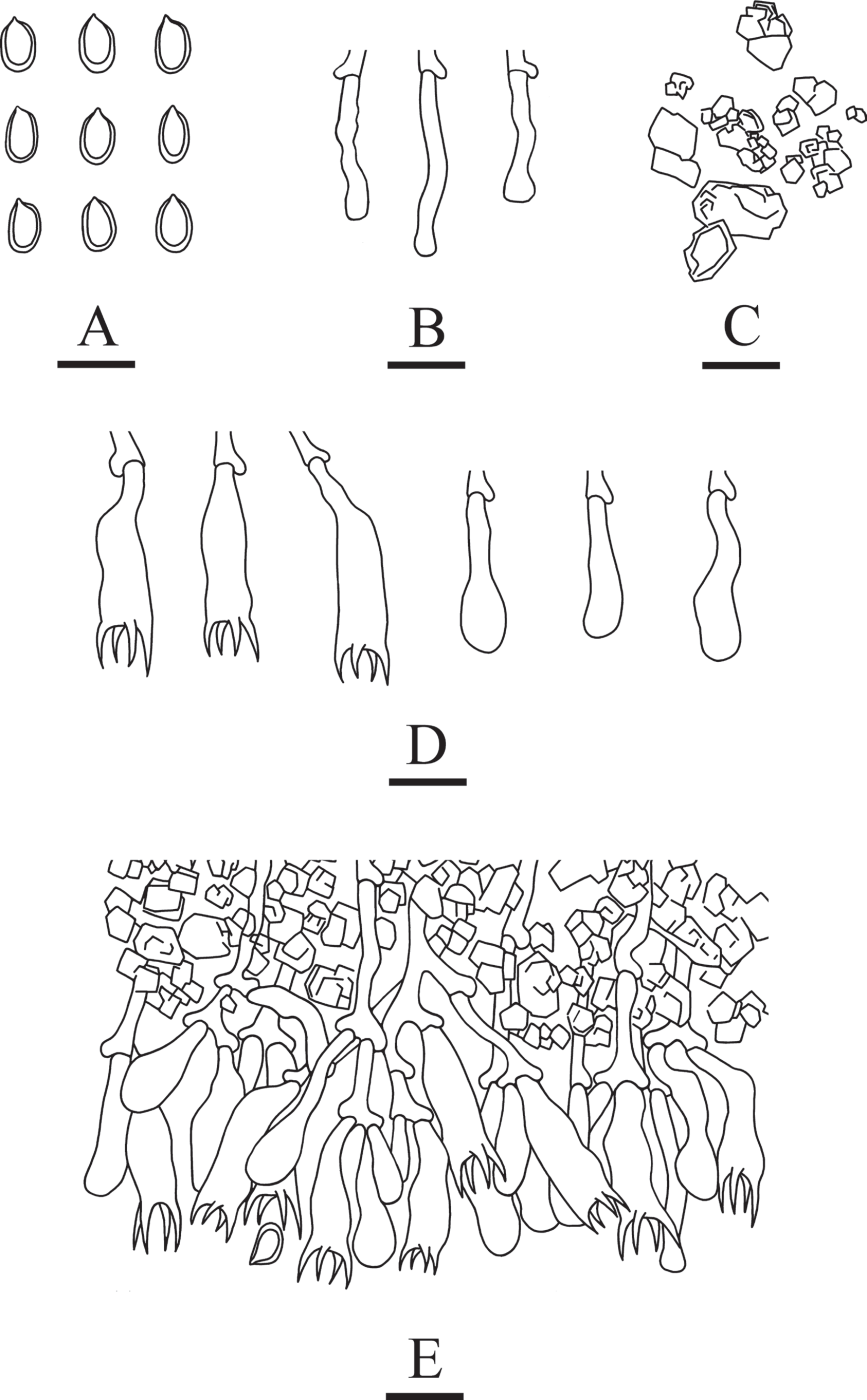
Microscopic structures of *Fissuracium ellipsoideum* (holotype, CLZhao 34584). **A**. Basidiospores; **B**. Cystidioles; **C**. Crystal; **D**. Basidia and basidioles; **E**. Part of the vertical section of hymenium. Scale bars: 10 μm (**A–E**).

**Figure 6.**
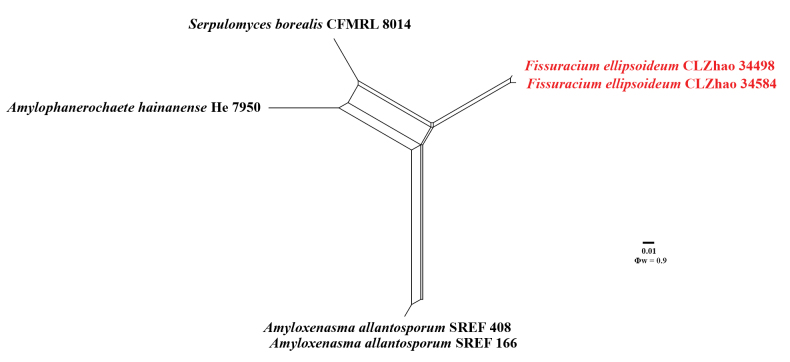
Split graphs showing the results of the PHI test for the ITS data of *Fissuracium ellipsoideum* and closely related taxa using LogDet transformation and splits decomposition. PHI test results, Φw ≤ 0.05, indicate significant recombination within the dataset. New taxa are in red.

##### Etymology.

*Ellipsoideum* (Lat.): refers to the ellipsoid basidiospores.

##### Basidiomata.

Annual, resupinate, adnate, membranaceous, fragile, without odor or taste when fresh, up to 20 cm long, 2 cm wide, and 150 μm thick. Hymenial surface smooth, obviously cracking, cream when fresh, turning to pale yellow upon drying. Sterile margin narrow, cream to pale yellow, up to 1 mm.

##### Hyphal structure.

Monomitic; generative hyphae with clamp connections, colorless, thin-walled, frequently branched, loosely interwoven, 1.3–2.7 µm in diameter, IKI–, CB–; tissues unchanged in KOH.

##### Hymenium.

Cystidia absent. Cystidioles clavate with a slightly swollen apex, 26.3 × 2.2 µm. Basidia more or less pyriform, slightly flexuous, attenuate toward the base, thin-walled, smooth, with four sterigmata and a basal clamp connection, 12.8–23.5 × 4.8–6.6 µm; basidioles pyriform, but smaller than basidia; subhymenial hyphae covered with smaller irregularly-shaped colorless crystals.

##### Basidiospores.

Ellipsoid, colorless, thick-walled, smooth, with one guttule, IKI–, slightly CB+, (4.7–)5–6.3(–6.4) × (3.2–)3.4–4.2(–4.4) µm, L = 5.73 µm, W = 3.85 µm, Q = 1.44–1.53 (n = 60/2).

##### Type of rot.

White rot.

##### Additional specimen (paratype) examined.

China • Yunnan Province: Diqing, Weixi County, Weideng Town, Songpo Village, GPS coordinates: 27°5'N, 99°13'E, elevation: 1,600 m asl., on the fallen angiosperm branch, leg. C.L. Zhao, 13 October 2023, CLZhao 34498 (SWFC 00034498).

## Discussion

The Amylocorticiales is a small order dominated by the wood-decaying fungi with corticioid basidiomata; samples of many species in the order are infrequently collected during investigations in China. In the present study, based on a combination of morphological features and molecular evidence, a new wood-inhabiting fungal genus, *Fissuracium* found in southwest China, is proposed.

Binder et al. (2010) constructed a six-loci nuclear dataset (nuc-ssu, nuc-lsu, 5.8S, *rpb1*, *rpb2* and *tef1*) for 191 species, which was analyzed with Maximum Parsimony, Maximum Likelihood and Bayesian Methods, which indicated that Amylocorticiales was the sister group of the Agaricales, suggesting that the greatest radiation of pileate-stipitate mushrooms resulted from the elaboration of resupinate ancestors. In the present study, *Fissuracium* forms a monophyletic clade within the family Amylocorticiaceae, which was clustered into the order Amylocorticiales. The molecular research indicated that the new genus *Fissuracium* formed a distinct lineage and was grouped with other branches, including *Amylophanerochaete* and *Serpulomyces*. However, morphologically, *Amylophanerochaete* differs from *Fissuracium* by having the pellicular to membranaceous basidiomata with rhizomorphs and amyloid basidiospores (Liu et al. 2026). *Serpulomyces* differs from *Fissuracium* by having merulioid hymenophores and cylindrical to fusiform basidiospores (Zmitrovich and Spirin 2002).

Amylocorticiaceae is a major group of wood-inhabiting fungi (Basidiomycota) characterized by relatively simple basidiomata and fewer diagnostic morphological features than those of polypores and mushrooms (Binder et al. 2010; Chen et al. 2020; Wu et al. 2022b). Despite this morphological simplicity, they exhibit higher species and phylogenetic diversity, yet remain substantially understudied (Floudas and Hibbett 2015; Miettinen et al. 2016; Justo et al. 2017; James et al. 2020; Zhao et al. 2024; Song et al. 2025). A substantial number of Amylocorticiaceae remain undocumented worldwide, particularly in subtropical and tropical ecosystems (Larsson 2007; Binder et al. 2010; Wu et al. 2022b; Mao et al. 2023; He et al. 2024; Liu et al. 2026; Zhou et al. 2024). As shown in this study, DNA sequence data are increasingly submitted to datasets, which support phylogenetic and evolutionary studies in this family. However, only the family Amylocorticiaceae accommodates all genera in this order, indicating that several lineages formed a distinct clade. Therefore, future taxonomic studies should continue to integrate detailed morphological assessments with multi-locus phylogenetic analyses to better document species diversity and to refine our understanding of the phylogeny and evolutionary history of this order.

## Supplementary Material

XML Treatment for Fissuracium

XML Treatment for Fissuracium ellipsoideum

## Additional information

### Artificial Intelligence (AI) use

The authors accept full responsibility for the content of the manuscript, including the disclosure of any use of AI.

No AI tools were used in the preparation of this manuscript.

### Author ORCIDs

C. Zhao https://orcid.org/0000-0002-8668-1075

Z. Gu https://orcid.org/0000-0003-1476-9040

X. Xu https://orcid.org/0009-0004-6000-1352

X. Liu https://orcid.org/0000-0003-0100-2094

### Data availability

All of the data that support the findings of this study are available in the main text.
